# Conservative Management of Splenic Injury in Blunt Abdominal Trauma: A Single Center Experience

**DOI:** 10.7759/cureus.43014

**Published:** 2023-08-06

**Authors:** Muhammad Osman, Muhammad Alam, Muhammad Iftikhar, Ali Gohar Khan

**Affiliations:** 1 Department of General, Benign Upper GI & Colorectal Surgery, Royal Bolton Hospital, Manchester, GBR; 2 Department of General Surgery, Hayatabad Medical Complex Peshawar, Peshawar, PAK; 3 Department of General Surgery, Fauji Foundation Hospital, Peshawar, PAK

**Keywords:** rta, splenic injury, non-operatively, conservative management, trauma

## Abstract

Background: Road traffic accidents are the greatest cause of death worldwide. Most intra-abdominal injuries caused by blunt abdominal trauma have been treated surgically for a very long period. Over the past few decades, conservative care has gained in popularity and effectiveness as a treatment choice for blunt abdominal trauma.

Objective: To determine the efficacy of conservative management in patients suffering from splenic injury in blunt abdominal trauma.

Methods: The study included 62 cases of blunt abdominal trauma treated non-operatively in the general surgery department of the Hayatabad Medical Complex Peshawar between June 2021 and December 2022.

Results: Minimal hemoperitoneum was observed in 47 (75.8%) cases, moderate hemoperitoneum was noted in 11 (17.7%) cases, and 4 (6.4%) patients didn’t have free fluid in the abdomen. There was no massive hemoperitoneum among the study patients. No major complications were observed during the study period. Only 7 (11.3%) cases develop minimal pleural effusion while 2 (3.2%) patients developed splenic abscess. Mortality was observed in only 1 (1.6%) case.

Conclusions: Conservative management is a safe and efficient strategy and should be considered as a first line of treatment for all hemodynamically stable patients who suffered blunt splenic injury.

## Introduction

In hemodynamically stable patients with blunt spleen injuries, conservative management is becoming the standard of care. As the spleen is a common organ injured by blunt abdominal trauma, splenectomy is usually carried out to prevent hemorrhagic shock. Due to the immune functions of the spleen, infections like post-splenectomy sepsis, malaria, and pneumonia can occur after a splenectomy [1]. Conservative treatment of splenic injury and other abdominal organs has become increasingly popular in recent decades [2].

Conservative management achieved good results in 80-90% of cases, and it is currently considered the primary treatment for spleen injuries in most centers, but there is no uniform protocol for this [3]. Arterial embolization in non-operative management of splenic trauma has been found and reported even after 8 hours of the injury. Conservative treatment has a success rate exceeding 90% and a failure rate as low as 8%, according to the literature [4,5].

As an important part of our immune system, the spleen filters and captures macrophages and cellular and non-cellular material such as pneumococcus, other bacteria, and red blood cells from the blood and plasma. A spleen should be salvaged because septicemia, opportunistic post-splenectomy infections, and malaria can occur after a splenectomy [1,6]. In blunt abdominal trauma, the physical examination and laboratory data are not specific for splenic injury [7]. A CT scan is important in this situation [8]. This study sought to determine the outcome and success rate of conservative management of splenic injury in blunt abdominal trauma.

## Materials and methods

A prospective and retrospective analysis of 62 cases of blunt abdominal trauma with contrast-enhanced computed tomography (CECT) scanned evidence of splenic injury, treated non-operatively between June 2021 and December 2022, was conducted. Ethical approval was granted by the ethical review board of Hayatabad Medical Complex Peshawar under reference #: 1210-1. After the initial resuscitation, hemodynamically stable patients and those whose hemodynamic status improved after initial resuscitation underwent CT scans of the abdomen. Following the administration of IV contrast, an abdominal CT scan was taken. On the basis of the CT scan appearance, splenic injuries were graded based on the organ injury scale. Primary and secondary assessments, as well as fluid resuscitation, were performed on all patients in accordance with early management recommendations for severe trauma.

Conservative management was considered for hemodynamically stable patients and those who became stable after initial resuscitation and had no signs of peritonitis clinically. A routine radiological examination of the cervical spine, chest, and pelvis was performed on all patients. In all cases in which a head injury was suspected, computed tomography scans of the head were performed.

A routine hemogram, serum biochemistry, and blood typing analysis were performed. Close monitoring of clinical and laboratory parameters was done in the intensive care unit (ICU). Pulse and blood pressure (BP) were monitored hourly. Hemoglobin and packed cell volume (PCV) were monitored every four hours. Clinical examinations were repeated at regular intervals to look for peritonitis symptoms developing in the abdomen. All patients were treated using intravenous fluids, nasogastric tube decompression, and nothing by mouth. Intravenous antibiotics were administered to patients who had contaminated external injuries.

Patients who experienced a drop in hemoglobin below 7 g/dL received blood transfusions. All patients were observed in the ICU for at least 48 hours before being transferred to the general surgical unit. Each patient underwent a radiological review with ultrasound scanning on the third and seventh days after admission as well as right before hospital discharge. Those patients who failed conservatively due to hemodynamic instability, a sustained decline in hemoglobin, or peritonitis underwent laparotomies. Data analysis was performed using Statistical Package for Social Sciences (SPSS), version 23.0 (IBM Corp., Armonk, NY).

## Results

**Figure 1:**
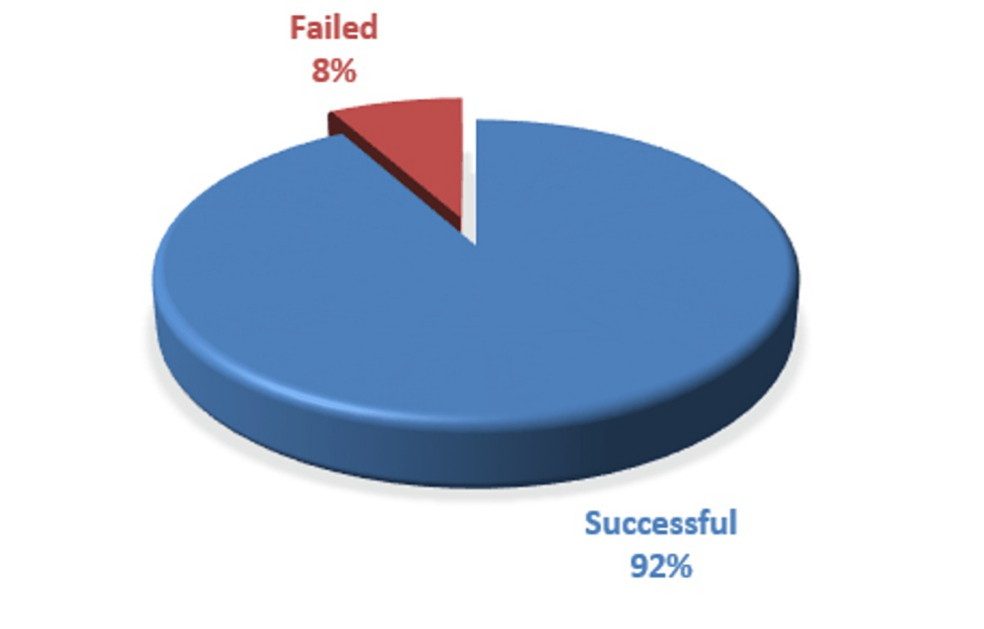
Success rate of conservative management

There were 47 (75.8%) males and 15 (24.2%) females. Out of 62 patients, 57 (91.9%) cases were treated successfully with conservative management, while 5 (8%) failed to non-operative management and were taken up for surgery (Figure 1).

Road traffic accidents were the most common cause of trauma at 33 (53.2%) cases, followed by 11 (17.7%) assault cases, with 10 (16.1%) cases having heavy objects falling on the abdomen, and 8 (12.9%) having fallen from a height (Table 1).

**Table 1:** Mechanism of injury

Mechanism	Frequency	Percentage
Road traffic accidents	33	53.2%
Assault	11	17.7%
Heavy object falling	10	16.1%
Fall from height	8	12.9%

The most common associate injury was lower rib fracture with hemothorax in 7 (11.3%) cases, followed by cervical fractures in 5 (8%), hepatic injury in 3 (4.8%), and renal injury in 1 (1.6%) case (Table 2).

**Table 2:** Common associated injury

Injury	Frequency	Percentage
Lower rib fracture	7	11.3%
Cervical fracture	5	8%
Hepatic injury	3	4.8%
Renal injury	1	1.6%

Based on the CT scan AAST grade I was noted in 12 (19.3%) cases, grade II in 27 (43.5%) cases, grade III in 17 (27.4%) cases, and grade IV in 6 (9.7%) cases. Minimal hemoperitoneum was observed in 47 (75.8%) cases, moderate hemoperitoneum was noted in 11 (17.7%) cases, and 4 (6.4%) patients didn’t have free fluid in the abdomen. There was no massive hemoperitoneum among the study patients (Table 3).

**Table 3:** Injury grade and other findings on CT scan

Findings	Frequency	Percentage
AAST grade		
Grade-I	12	19.3%
Grade-II	27	43.5%
Grade-III	17	27.4%
Grade-IV	6	9.7%
Other findings		
Minimal hemoperitoneum	47	75.8%
Moderate hemoperitoneum	11	17.7%
No free fluid	4	6.4%
Major hemoperitoneum	0	0%

No major complications were observed during the study period. Only 7 (11.3%) cases developed minimal pleural effusion while 2 (3.2%) patients developed splenic abscess. Mortality was observed in only 1 (1.6%) case due to moderate hemoperitoneum (Table 4).

**Table 4:** Complications and mortality

Complications	Frequency	Percentage
Minimal pleural effusion	7	11.3%
Splenic abscess	2	3.2%
Mortality	1	1.6%

Grade I-II patients were discharged by 5 days and grades III-IV by 12 days (mean stay 8.5 days). ICU stay ranged between 2-4 days (mean 3 days).

## Discussion

In blunt abdominal trauma, the spleen is the most commonly injured intra-abdominal organ. In past decades, laparotomy was regarded as the preferred method of treatment for blunt splenic injury. As a result of King H & Schumacher HB's early description of severe post-splenectomy infection, conservative treatments for the splenic injury were considered [9]. Over time, solid organ injury management has evolved with the primary objective of reducing mortality and morbidity from hemorrhagic shock and sepsis.

Compared with earlier trials, which mostly involved pediatric patients, conservative management has been shown to be effective in adults in recent years. For patients with stable hemodynamics, conservative management is currently the preferred treatment for splenic injury. Several studies have been conducted on the conservative management of abdominal trauma [10]. Blunt abdominal trauma may be managed conservatively with a wide range of success rates.

CT of the abdomen is the most effective non-operative approach to abdominal trauma. Stawicki et al. described his initial evaluations of CT in guiding the nonsurgical management of abdominal trauma [11]. If the hemoperitoneum is less than 250 ml and the patient is hemodynamically stable, conservative management should be considered. Initially, conservative approaches were limited to minor injuries (grades I and II) but were sometimes extended to grades III and IV. In the following years, many trauma experts suggested that hemodynamic stability is more important than the grade of injury when assessing blunt abdominal trauma. This concept was supported by Costa G et al., who described conservative management as a means of managing grade IV-V injuries [12].

The majority of patients were classified as grade II or III according to the literature review [13]. The risk of failure increased with increasing grades of injury. In our study only 6 (9.7%) patients suffered grade IV injuries. No grade V injury was reported, while 56 (90.3%) cases had grade I, II, and III injuries, which is in line with international literature.

According to Hsieh et al., the amount of free fluid in the abdomen did not predict treatment failure, and other studies have confirmed this [14]. In our series, 11 (17.7%) had moderate amounts of free fluid in the abdomen. No large amount or major hemoperitoneum was observed in the failed cases. Therefore, moderate to major hemoperitoneum no longer indicates failure.

The possibility of missed hollow viscous injuries is one of the challenges of conservative management. A study by Fischer et al. found that 15% of adults with abdominal trauma have gastrointestinal disruption [15]. In adults with blunt abdominal trauma, routine conservative management is not recommended due to the high incidence of missed hollow viscous injuries. Despite this, several studies demonstrated the effectiveness of conservative management [16,17]. Clinical examination and a quality radiological assessment are the most effective ways to detect hollow viscous injuries. Consequently, patients with high spinal cord injuries may not show classical abdominal signs at the time of clinical examination and may take up to four days to be diagnosed. Pneumoperitoneum, free fluid without solid organ injury, thickened small intestines and dilated small intestines are common CT findings of hollow viscous injuries. However, radiological imaging should not be blindly trusted, as this can lead to dangerous outcomes.

The conservative management of blunt abdominal trauma has revolutionized patient care. It has a wide range of success rates. One possible explanation for this variation is differences in surgical practice between institutions. The success rate depends unquestionably on how often it is applied. Conservative management of lower grades blunt abdominal trauma injuries would likely have a high success rate.

## Conclusions

In most patients who sustained blunt abdominal trauma with solid organ injury, conservative management is safe and effective. Hemodynamic stability plays a crucial role in the successful management of blunt abdominal trauma. An essential part of treatment is to repeat clinical examinations in order to detect missed hollow viscous injury. In unstable patients, conservative management is not recommended.
